# Association between APE1 rs1760944 and rs1130409 polymorphism with prostate cancer risk: A systematic review and meta-analysis

**DOI:** 10.1097/MD.0000000000027630

**Published:** 2021-11-19

**Authors:** Jinnian Liu, Jian Zheng, Yu Guo, Xia Sheng, Yongjian Yin, Shengqiang Qian, Bin Xu, Wei Xiong, Xiangrui Yin

**Affiliations:** a Department of Urology, Traditional Chinese Medicine Hospital, Chongqing, China; b Department of Urology, Second People's Hospital of Banan District, Chongqing, China.

**Keywords:** APE1 rs1130409, APE1 rs1760944, polymorphism, prostate cancer

## Abstract

**Background:**

Recently, some studies have suggested that the association of apurinic/apyrimidinic endonuclease 1 (APE1) gene polymorphism with prostate cancer (PCa) risk, but there are still some controversies. Hence, we elaborated the relationship between APE1 rs1760944 and rs1130409 gene and PCa risk through systematic literature review and meta-analysis.

**Methods:**

As of March 2020, EMBASE, PubMed, the Cochrane Library, Science Direct/Elsevier, MEDLINE and CNKI were used for systematic literature retrieval to investigate the correlation between APE1 rs1760944 and rs1130409 gene polymorphism with PCa risk. Meta-analysis was performed using Review Manager and Stata software.

**Results:**

Seven studies were distinguished, consists of 1769 cases of PCa patients and 2237 normal controls. Our results illustrated that there are significant correlation between the APE1 rs1760944 gene polymorphism and PCa in all genetic models (*P* < .05). The combined odds ratios and 95% confidence intervals were as follows: Additive model (ORs 0.62, 95%, CI [0.39, 0.97]); Codominant model (ORs 0.74, 95% CI [0.58, 0.95]); Dominant model (ORs 0.75, 95%, CI [0.59, 0.95]); Recessive model (ORs 0.63, 95% CI [0.41, 0.96]); Allele model (ORs 0.78, 95% CI [0.65, 0.94]). There also have significant associations between APE1 rs1130409 polymorphisms and PCa in all genetic models (*P* < .05). The combined odds ratios and 95% confidence intervals were as follows: Additive model (ORs 1.37, 95%, CI [1.01, 1.85]); Codominant model (ORs 1.21, 95% CI [1.01, 1.44]); Dominant model (ORs 1.33, 95%, CI [1.02, 1.73]); Recessive model (ORs 1.74, 95% CI [1.06, 2.85]); Allele model (ORs 1.14, 95% CI [1.00, 1.29]).

**Conclusion:**

This study suggests that APE1 rs1760944 polymorphisms might be a protective factor of PCa, and APE1 rs1130409 is suggested to be a risk factor of PCa. APE1 rs1760944 and rs1130409 polymorphisms may be used in the risk assessment of PCa.

## 1 Introduction

Prostate cancer (PCa) is one of the most common tumors in men, causing more than 250,000 deaths every year.^[1,2]^ At present, the etiology and pathogenesis of PCa are still unclear.^[3,4]^ Therefore, it is of great significance to identify the risk factors of PCa for the development of intervention and protection measures. Currently, much evidence suggests that complex interactions between genetic and environmental factors are involved in the development of PCa.^[5–8]^ A genome-wide association study (GWAS) found that at least 35 loci were associated with PCa.^[6,7]^ About 42% of PCa risk can be explained by genetic factors. It is of great value and significance to test these risk alleles in the population.^[8]^

The difference of DNA repair ability (DRC) among individuals is an important factor affecting individual's cancer risk.^[9]^ DNA repair gene polymorphism may cause this variation. Apurinic/apyrimidinic endonuclease 1 (APE1) in human is located on chromosome 14q11.2, which consists of five exons and spans about 2.5 to 3 kb of DNA.^[10,11]^ In addition to its role in DNA repair, APE1 is also known as a transcriptional coactivator of many transcription factors, such as AP-1 HIF-1 α, p53 and NF-κB, which are involved in cancer promotion and progression.^[12]^ APE1 is a promising target for pharmacological treatment of some cancer types.^[13]^ To date, epidemiological studies have shown that single nucleotide polymorphisms (SNPs) in APE1 may confer an individual's susceptibility to PCa.^[14,15]^ Some related studies have reported the correlation between the SNP variation of APE1 rs1760944 and rs1130409 and PCa risk, but the results on this issue are contradictory. Some studies reported that this polymorphism may increase the risk of PCa, but other studies did not.^[16–22]^ Therefore, we systematically reviewed the available literature and performed a meta-analysis to evaluate the the relationship between APE1 rs1760944 and rs1130409 gene polymorphisms and PCa risk, which may provide valuable insights for the risk assessment of PCa.

## 2 Materials and methods

### 2.1 Literature search

Published studies on the association between APE1 rs1760944 and rs1130409 gene polymorphisms and PCa risk were restricted to a meta-analysis. Two independent researchers retrieved documents from Science Direct/Elsevier, Embase, CNKI, PubMed, the Cochrane Library and Medline with a search deadline of March 2020 and no language or learning type restrictions. The retrieval terms consisted of MeSH terms and text words. For example, the retrieval terms for the APE1 rs1760944 and rs1130409 gene were “APE1 rs1760944 gene ”, “rs1760944 ”, “APE1 rs1130409 gene”, “rs1130409”, “Apurinic/apyrimidinic (AP) endonuclease”, or “APEX1 gene”; those for PCa were “prostate cancer,” “prostatic neoplasms”, “cancer of prostate”, “cancer of the prostate”, “neoplasms, prostate”, “neoplasms, prostatic”, “prostate neoplasms”, “prostatic cancer”, or “PCa”; and those for the polymorphism were “SNP”, “single-nucleotide polymorphism”, “polymorphism”, “variation”, or “mutation”. All abstracts and relevant documents were retrieved. At the same time, the references in related articles are manually searched, and only full-text documents were included. This study was approved by the Ethics Committee of Chongqing Traditional Chinese Medicine Hospital.

### 2.2 Eligibility criteria

Inclusion criteria: If case-control studies tested the relationship between APE1 rs1760944 and rs1130409 gene variants and PCa, the study was included. The case group was PCa patients The criteria for PCa were suspicious findings on a digital rectal examination (DRE) and/or elevated PSA serum levels (>4.0 ng/ml), followed by histopathological confirmation of prostate cancer. And the control groups were normal people. The SNPs of APE1 rs1760944 and rs1130409 were genotyped by polymerase chain reaction-restriction fragment length polymorphism. Effective data were extracted from the article, including eligible and genotype cases and controls, as well as the number of cases and controls of each APE1 rs1760944 and rs1130409 genotype.

Exclusion criteria: Case reports, only abstracts, meeting reports, and studies lacking a control population were excluded. Additionally, literature reviews that duplicated previous publications were excluded.

### 2.3 Study selection and validity assessment

Two independent researchers searched the titles and abstracts of the literature, and all relevant studies that met the research criteria were screened. If the title and abstract of the literature could not be used to judge whether the study should be included, the full text was retrieved for analysis. If there were differences in the literature included, it was assessed by consensus or by a third reviewer. In meta-analysis, two reviewers conducted quality assessments based on the main criteria for nonrandomized and observational studies of the Newcastle-Ottawa Quality Assessment scale (NOS).

### 2.4 Data extraction and statistical analysis

Data were collected from the literatures by three reviewers and contained demographic statistics (authors, publishing year, nation, number and average age of participants) and number of cases and controls for each APE1 rs1760944 and rs1130409 genotype. Differences were settled by consensus. Two researchers conducted quantitative meta-analyses using RevMan and Stata software. The combined odds ratio (OR) and its 95% confidence interval (CI) were calculated. Heterogeneity was assessed using p-values and the I-square statistic (I^2^) in the pooled analysis, which represents the percentage of total variation in all studies. If the *P* value is less than 0.1 or the I^2^ value is more than 50%, the random effect model is used to analyze and summarize the estimated values. Otherwise, the fixed effect model is applied. The association between APE1 rs1760944 and rs1130409 polymorphisms and the risk of PCa was studied in an allele model (G vs T) (Both elements of each subject's genotype are considered to correspond to the same value of the dependent variable. The associations with these individual alleles are then tested.), additive model (GG vs TT) (testing is designed specifically to reveal associations that depend additively upon the minor allele – that is, where having two minor alleles (GG) rather than having no minor alleles (TT)) is twice as likely to affect the outcome in a certain direction as is having just one minor allele (TG) rather than no minor alleles (TT)), dominant model (GG and TG vs TT) (This model specifically tests the association of having at least one minor allele G (either TG or GG) vs not having it at all (TT)), recessive model (GG vs TG and GG) (This model specifically tests the association of having the minor allele D as both alleles (DD) vs having at least one major allele G (TG or TT)), and codominant model (TG vs TT) (testing on the genotypes TT, GG, and TG without regard to any “order” or allelic count or allelic pairing that they might have). The *P* < .05 suggest significant associations between the APE1 rs1760944 and rs1130409 gene polymorphism and PCa. In addition, publication bias was detected by visual symmetry of funnel plots, with asymmetry suggesting possible publication bias. This was also assessed by the Begg's and Egger's test in the meta-analysis. If the *P*-value was less than .05, publication bias existed.

## 3 Results

### 3.1 Characteristics of the included studies

**Figure 1:**
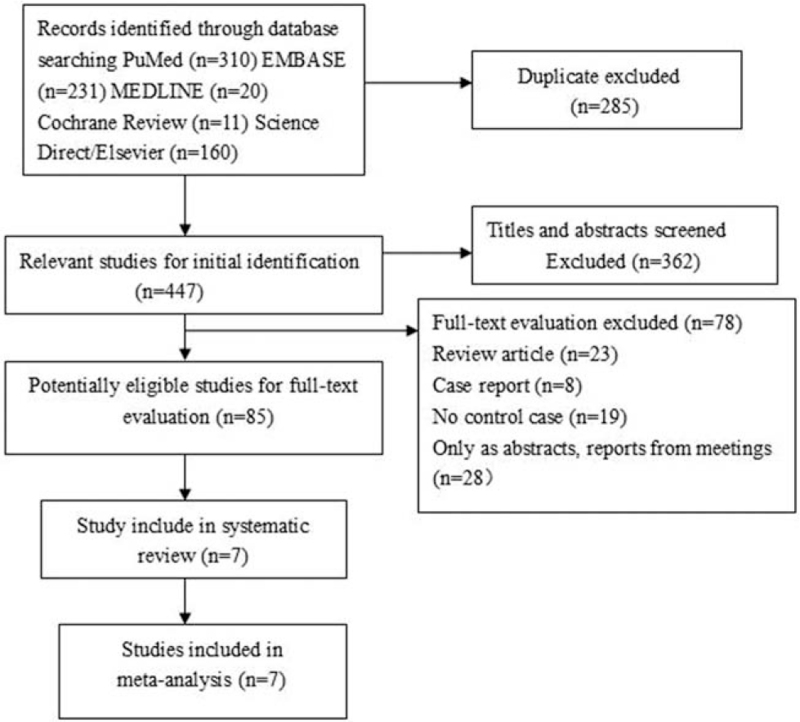
Flow diagram of the selection of eligible studies.

The detailed check procedure is exhibited in Figure 1. A total of447 unduplicated studies were reviewed. seven studies were ultimately screened out. Table 1 summarizes the data from the seven studies. All retrieved researches involved 1769 cases of PCa patients and 2237 normal controls. All these studies reported exclusion/inclusion criteria.^[16–22]^

**Table 1:** Characteristics of the included studies.

		Case						Control						
Author	Country	N	T	G	TT	TG	GG	N	T	G	TT	TG	GG	genotype
Lan C 2006^a^	America	124	148	101	42	64	17	116	143	81	42	59	11	rs1130409
Lan C 2006^b^	America	228	252	204	65	122	41	219	254	180	73	108	36	rs1130409
Lan C 2006^c^	America	124	219	29	98	23	3	116	194	38	82	30	4	rs1760944
Lan C 2006^d^	America	228	431	25	204	23	1	219	403	33	186	31	1	rs1760944
Kuasne H 2011	Brazil	172	251	93	84	83	5	172	276	68	106	64	2	rs1130409
Lavender NA 2010	America	208	252	120	82	88	16	665	817	445	274	269	88	rs1130409
Mandal RK 2012	India	192	283	101	106	71	15	224	330	118	118	94	12	rs1760944
Mittal RD 2012	India	195	288	104	108	72	15	250	373	127	136	101	13	rs1130409
Jing B 2013	China	198	228	147	78	93	27	156	170	142	47	76	33	rs1760944
Pournourali M 2015	Iran	100	90	110	15	60	25	100	110	90	30	50	20	rs1130409

Four studies explored the relationship between APE1 rs1760944 gene polymorphism and PCa, And six studies have studied the relationship between APE1 rs1130409 gene polymorphism and PCa. In addition, restriction fragment length polymorphism analysis was used to detect APE1 gene polymorphism in all these studies.

### 3.2 Meta-analysis

**Figure 2:**
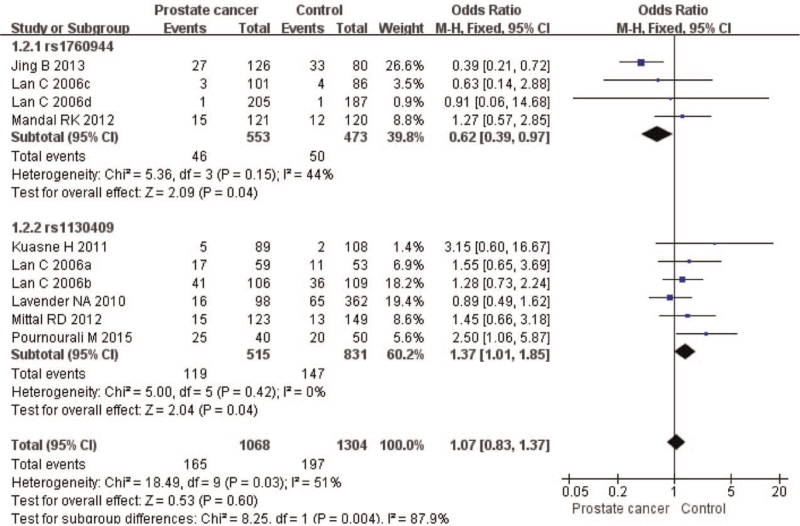
Forest plot showing the meta-analysis outcomes of the additive model.

**Figure 3:**
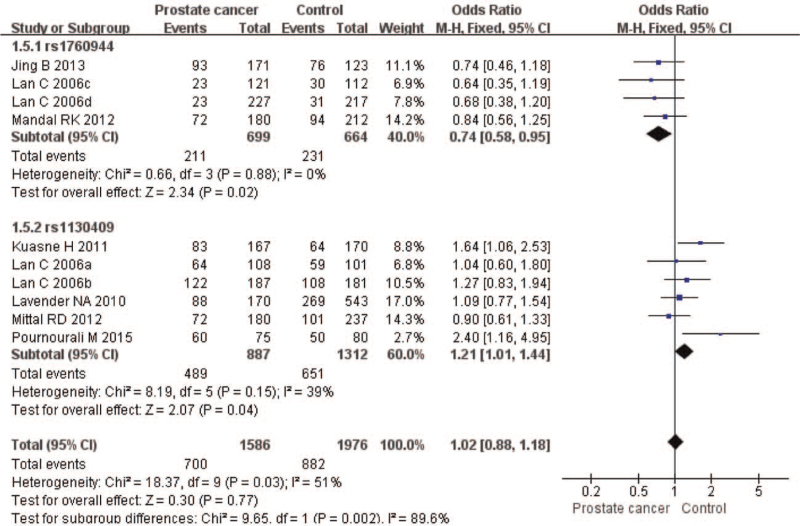
Forest plot showing the meta-analysis outcomes of the codominant model.

**Figure 4:**
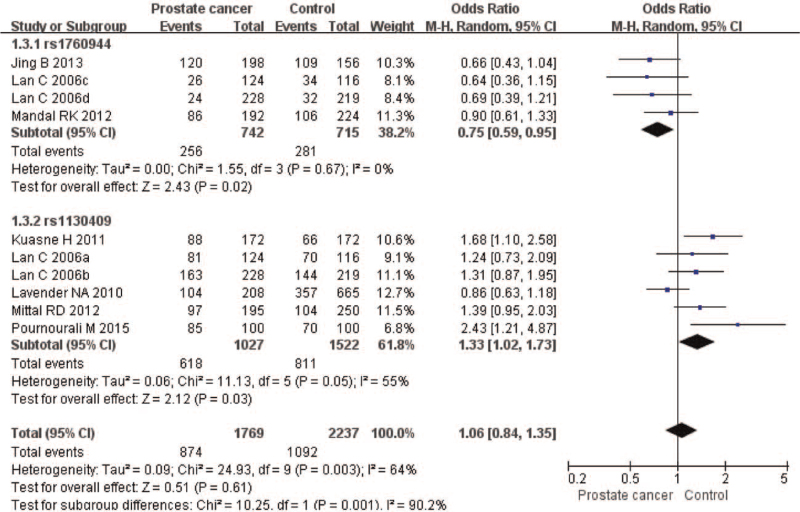
Forest plot showing the meta-analysis outcomes of the dominant model.

**Figure 5:**
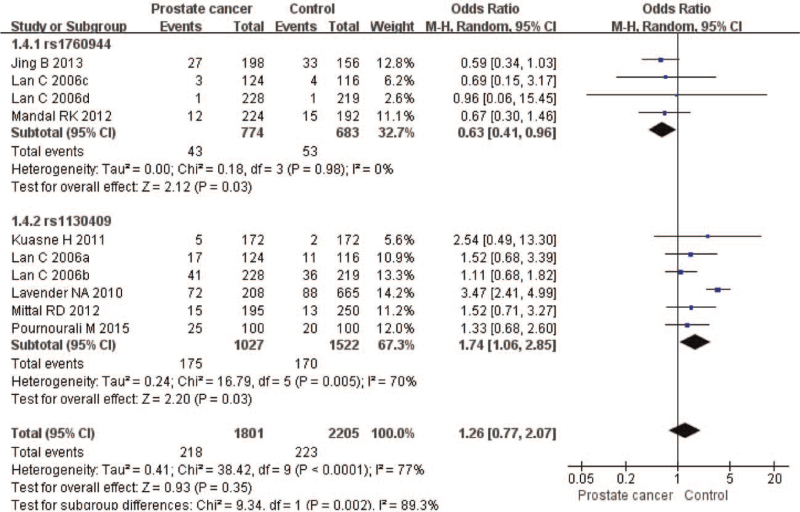
Forest plot showing the meta-analysis outcomes of the recessive model.

**Figure 6:**
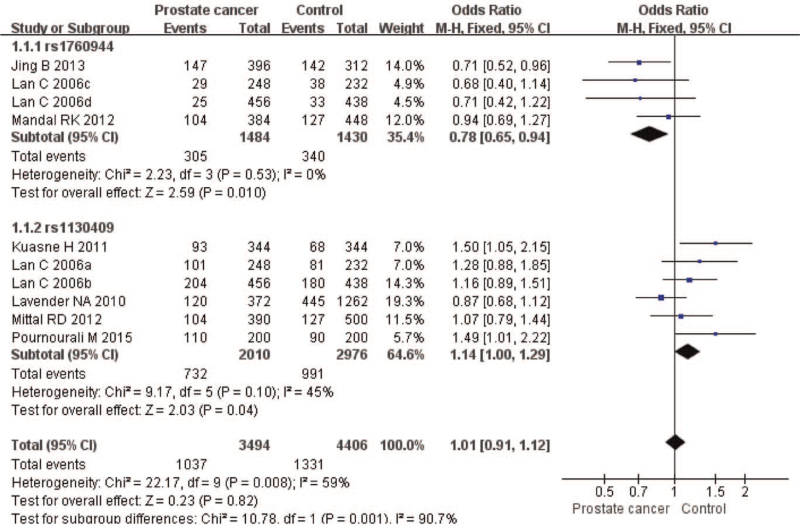
Forest plot showing the meta-analysis outcomes of the allele model.

**Figure 7:**
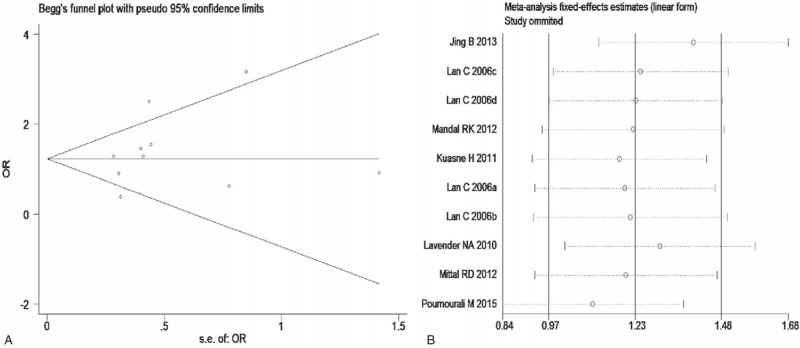
Begg's publication bias and Sensitivity analysis plot of Additive model, (A) Begg's publication bias; (B) Sensitivity analysis.

**Figure 8:**
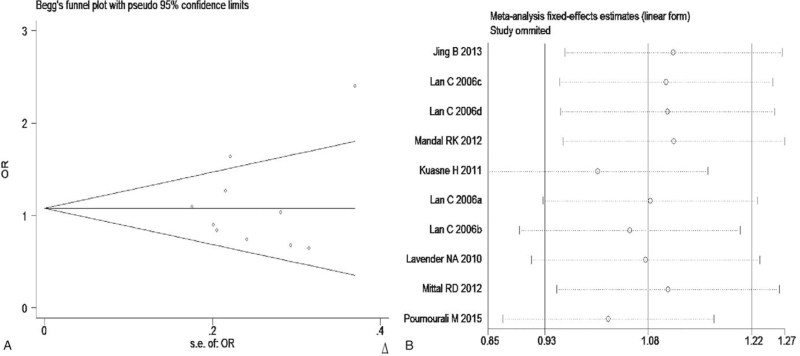
Begg's publication bias and Sensitivity analysis plot of Codominant model, (A) Begg's publication bias; (B) Sensitivity analysis.

**Figure 9:**
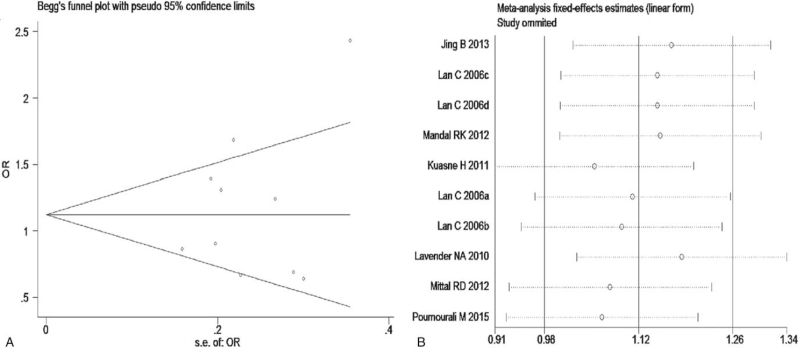
Begg's publication bias and Sensitivity analysis plot of Dominant model, (A) Begg's publication bias; (B) Sensitivity analysis.

**Figure 10:**
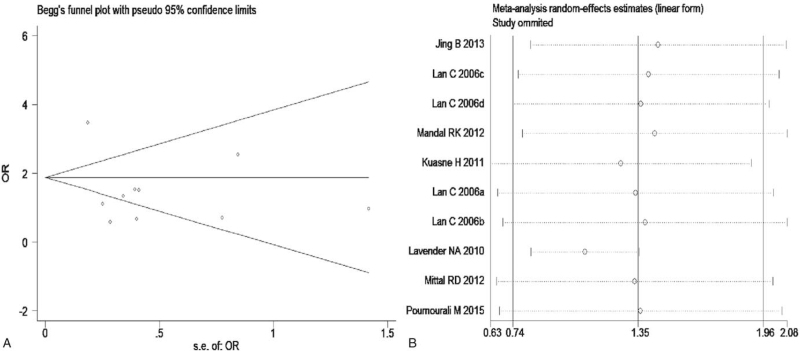
Begg's publication bias and Sensitivity analysis plot of Recessive model, (A) Begg's publication bias; (B) Sensitivity analysis.

**Figure 11:**
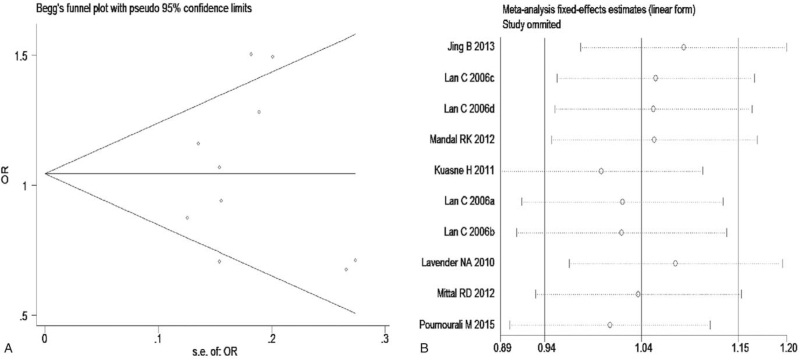
Begg's publication bias and Sensitivity analysis plot of Allele model, (A) Begg's publication bias; (B) Sensitivity analysis.

The meta-analysis revealed significant associations between the APE1 rs1760944 gene polymorphism and PCa in all genetic models (*P* < .05). Suggests that APE1 rs1760944 polymorphisms might be a protective factor of PCa, The combined ORs and 95% CIs were as follows: Additive model (ORs 0.62, 95%, CI [0.39, 0.97]) (Fig. 2); Codominant model (ORs 0.74, 95% CI [0.58, 0.95]) (Fig. 3); Dominant model (ORs 0.75, 95%, CI [0.59, 0.95]) (Fig. 4); Recessive model (ORs 0.63, 95% CI [0.41, 0.96]) (Fig. 5); Allele model (ORs 0.78, 95% CI [0.65, 0.94]) (Fig. 6). There also have significant associations between APE1 rs1130409 polymorphisms and PCa in all genetic models (*P* < .05). APE1 rs1130409 is suggested to be a risk factor of PCa. The combined odds ratios and 95% confidence intervals were as follows: Additive model (ORs 1.37, 95%, CI [1.01, 1.85]) (Fig. 2); Codominant model (ORs 1.21, 95% CI [1.01, 1.44]) (Fig. 3); Dominant model (ORs 1.33, 95%, CI [1.02, 1.73]) (Fig. 4); Recessive model (ORs 1.74, 95% CI [1.06, 2.85]) (Fig. 5); Allele model (ORs 1.14, 95% CI [1.00, 1.29]) (Fig. 6). Begg's funnel plots were largely symmetric (Figures 7A, 8A, 9A,10A, 11A), suggesting that there was no publication bias in the meta-analysis. Egger's regression test also indicated little evidence of publication bias in all genetic models (*P* > .05) (Table 2). We also conducted a sensitivity analysis of the meta-analysis. We omitted one study at a time, and the calculated combined ORs for the remaining studies yielded consistent results. In the overall meta-analysis, no single study significantly changed the combined results, which indicated that the results were statistically stable and reliable (Figures 7B, 8B, 9B, 10B, 11B).

**Table 2:** Egger's test of publication bias.

	Coeff.	Std. err.	*t*	*P*>|*t*|	[95% Conf. interval]
Allele	0.40	2.41	0.17	.87	−5.17 5.97
Additive	1.45	1.37	1.05	.32	−1.72 4.62
Dominant	−2.56	1.83	−1.40	.20	−6.77 1.65
Recessive	2.23	2.91	0.77	.46	−4.49 8.95
Codominant	1.56	2.79	0.56	.59	−4.86 7.99

## 4 Discussion

In this study, we focused on seven studies reported to elucidate the relationship between APE1 rs1760944 and rs1130409 gene polymorphisms and PCa risk. Four studies explored the relationship between APE1 rs1760944 gene polymorphism and PCa, the data indicate that APE1rs1760944 gene polymorphism might a protective factor of PCa. For APE1 rs1130409, six studies have studied the relationship between APE1 rs1130409 gene polymorphism and PCa. However, the data indicate that APE1 rs1130409 gene polymorphism might one of the risk factors of PCa.

Human exposure to a variety of environmental carcinogens and endogenous/exogenous reactive oxygen species may lead to DNA damage, which ultimately increases the susceptibility of men to prostate cancer (PCa).^[23,24]^ The body's complex DNA repair system has evolved to correct DNA damage caused by the environment.^[25]^ There are many DNA repair pathways, each responsible for repairing specific types of damage.^[26]^ The base excision repair (BER) pathway mainly eliminates DNA damage caused by ionizing radiation, reactive oxygen radicals and methylation agents.^[27]^ Apurinic/apyrimidinic (AP) endonuclease APE1 gene is located at chromosome 14q11.2, which encodes one of the main enzymes in BER pathway and accounts for almost all the cleavage activity of abasic sites observed in cell extract.^[28–32]^ In addition, APE1 is also a multifunctional protein, which participates in other important cell processes, including response to oxidative stress, regulation of transcription factors, cell cycle control and apoptosis. Therefore, the abnormal expression of APE1 gene may lead to the repair of defects in these lesions and give individuals the susceptibility to cancer.^[33–38]^ Epidemiological studies have found that APE1 gene polymorphism is related to the risk of lung cancer, breast cancer, colorectal cancer and bladder cancer.^[35,36]^ 18 SNPs were identified in APE1, including two functional SNPs rs1760944 in the promoter region and rs1130409 in the fifth exon. These two functional SNPs have been widely studied. It is reported that APE1 rs1760944 T > G polymorphism is related to the change of promoter activity in vitro, compared with T allele, rs1760944 G allele is related to reducing cancer risk by enhancing transcription activity. APE1 rs1130409 polymorphism T to G, in the biological significance, APE1 codon T to G polymorphism is associated with the delay of lymphocyte mitosis in healthy subjects, suggesting a higher sensitivity to ionizing radiation.^[39]^ And some authors describe the relationship between allele G and prostate cancer.^[40]^ Some studies have analyzed this transition and support this polymorphism as a low penetrance risk factor for cancer development.^[41]^

Our findings suggest that APE1 rs1760944 gene polymorphism might be a protective factor of prostate cancer. In this meat-analysis the G/G genotype in control group was significantly higher than prostate cancer group. The transformation from codon T to G at this site, may influenced the enhancing the transcriptional activity, protect DNA from damage, in turns reduce gene mutations, resulting in the decrease risk of prostate cancer. For APE1 rs1130409, our findings suggest that APE1 rs1130409 might a risk factor of prostate cancer, in our meta analysis, G/G genotype in prostate cancer group was significantly higher than in control. The possible mechanisms may the APE1 codon T transversion G polymorphism is have higher sensitivity to ionizing radiation, G alleles are more likely to cause gene damage, resulting in the increase risk of prostate cancer. However, studies with larger sample sizes are needed to better illuminate the mechanisms of the APE1 rs1760944 and rs1130409 in the prostate cancer tumorigenesis.

There are some limitations in our study, which need to be taken into consideration when interpreting the results of this meta-analysis. First, the sample size of each study was relatively small, and a total of 1769 prostate cancer patients and 2237 normal controls were investigated in the seven studies. Second, several studies on this issue were excluded owing to a lack of control data. Furthermore, because of the limited amount of original research, a subgroup of APE1 rs1760944 and rs1130409 gene polymorphism in different race was not conducted. As such, it is difficult to draw definitive conclusions about the clinical value of APE1 rs1760944 and rs1130409 gene variants inPCa.

In summary, the results of this meta-analysis suggest that the current article adds to the evidence of an association between the APE1 gene polymorphisms and prostate cancer progression. These data suggest that APE1 rs1760944 polymorphisms might be a protective factor of prostate cancer, and APE1 rs1130409 is suggested to be a risk factor of prostate cancer. APE1 rs1760944 and rs1130409 polymorphisms may be used in the risk assessment of PCa. However, studies with larger sample sizes are needed to definitively determine the correlation between APE1 rs1760944 and rs1130409 gene polymorphisms and prostate cancer.

## Author contributions

**Conceptualization:** Jinnian Liu, Jian Zheng, Yongjian Yin, Shengqiang Qian, Bin Xu, Wei Xiong, Xiangrui Yin.

**Data curation:** Jinnian Liu, Yu Guo, Xia Sheng, Yongjian Yin, Shengqiang Qian, Bin Xu, Xiangrui Yin.

**Formal analysis:** Jinnian Liu, Jian Zheng, Xia Sheng, Yongjian Yin, Wei Xiong, Xiangrui Yin.

**Funding acquisition:** Jinnian Liu, Jian Zheng, Yu Guo, Xia Sheng, Shengqiang Qian, Bin Xu, Wei Xiong, Xiangrui Yin.

**Investigation:** Jinnian Liu, Jian Zheng, Xia Sheng, Xiangrui Yin.

**Methodology:** Jinnian Liu, Yu Guo, Shengqiang Qian, Bin Xu, Wei Xiong, Xiangrui Yin.

**Project administration:** Jinnian Liu, Yu Guo, Xia Sheng, Yongjian Yin, Shengqiang Qian, Bin Xu, Wei Xiong, Xiangrui Yin.

**Resources:** Jinnian Liu, Jian Zheng, Xia Sheng, Bin Xu, Xiangrui Yin.

**Software:** Jian Zheng, Xia Sheng, Xiangrui Yin.

**Supervision:** Jian Zheng, Yu Guo, Yongjian Yin, Shengqiang Qian, Wei Xiong, Xiangrui Yin.

**Validation:** Jian Zheng, Yongjian Yin, Shengqiang Qian, Xiangrui Yin.

**Visualization:** Jinnian Liu, Yu Guo, Xia Sheng, Shengqiang Qian, Wei Xiong, Xiangrui Yin.

**Writing – original draft:** Jinnian Liu, Yu Guo, Xia Sheng, Yongjian Yin, Wei Xiong, Xiangrui Yin.

**Writing – review & editing:** Jinnian Liu, Jian Zheng, Yu Guo, Yongjian Yin, Xiangrui Yin.

## References

[1] Ferlay J Shin H Bray F. Estimates of worldwide burden of cancer in 2008: GLOBOCAN 2008. Int J Cancer 2010;127:2893–917. 21351269 10.1002/ijc.25516

[2] Crawford ED. Understanding the epidemiology, natural history, and key pathways involved in prostate cancer. Urology 2010;73:04–10. 10.1016/j.urology.2009.03.001 19375626

[3] Jemal A Center MM DeSantis C. Global patterns of cancer incidence and mortality rates and trends. Cancer Epidemiol Biomarkers Prev 2010;19:1893–907. 20647400 10.1158/1055-9965.EPI-10-0437

[4] Horwich A Parker C Bangma C. Prostate cancer: ESMO Clinical Practice Guidelines for diagnosis, treatment and follow-up. Ann Oncol 2010;21:129–33. 10.1093/annonc/mdq174 20555062

[5] Chung CC Ciampa J Yeager M. Fine mapping of a region of chromosome 11q13 reveals multiple independent loci associated with risk of prostate cancer. Hum Mol Genet 2011;20:2869–78. 21531787 10.1093/hmg/ddr189 PMC3118760

[6] Sun J Purcell L Gao Z. Association between sequence variants at 17q12 and 17q24.3 and prostate cancer risk in European and African Americans. Prostate 2008;68:691–7. 18361410 10.1002/pros.20754 PMC3176499

[7] Thomas G Jacobs KB Yeager M. Multiple loci identified in a genome-wide association study of prostate cancer. Nat Genet 2008;40:310–5. 18264096 10.1038/ng.91

[8] Waters KM Le Marchand L Kolonel LN. Generalizability of associations from prostate cancer genome-wide association studies in multiple populations. Cancer Epidemiol Biomarkers Prev 2008;18:1285–9. 10.1158/1055-9965.EPI-08-1142 PMC2917607 19318432

[9] Berwick M Vineis P. Markers of DNA repair and susceptibility to cancer in humans: an epidemiologic review. J Natl Cancer Inst 2000; 2000;92:874–97. 10.1093/jnci/92.11.874 10841823

[10] Xi T Jones IM Mohrenweiser HW. Many amino acid substitution variants identified in DNA repair genes during human population screenings are predicted to impact protein function. Genomics 2004;83:970–9. 15177551 10.1016/j.ygeno.2003.12.016

[11] Li M Wilson DM 3rd. Human apurinic/apyrimidinic endonuclease 1. Antioxid Redox Signal 2013;Aug 20. 10.1089/ars.2013.5492 PMC3901322 23834463

[12] Tell G Damante G Caldwell D Kelley MR. The intracellular localization of APE1/Ref-1: more than a passive phenomenon? Antioxid Redox Signal 2005;7:367–84. 15706084 10.1089/ars.2005.7.367

[13] Tell G Fantini D Quadrifoglio F. Understanding different functions of mammalian AP endonuclease (APE1) as a promising tool for cancer treatment. Cell Mol Life Sci 2010;67:3589–608. 20706766 10.1007/s00018-010-0486-4 PMC11115856

[14] Wu B Liu HL Zhang S. Lack of an association between two BER gene polymorphisms and breast cancer risk: a meta-analysis. PLoS One 2012;7:e50857. 23272074 10.1371/journal.pone.0050857 PMC3522727

[15] Gu D Wang M Zhang Z. The DNA repair gene APE1 T1349G polymorphism and cancer risk: a meta-analysis of 27 case-control studies. Mutagenesis 2009;24:507–12. 16-22. 19762350 10.1093/mutage/gep036

[16] Pournourali M Tarang AR Yousefi M. The association between 1349T.G polymorphism of ApE1 gene and the risk of prostate cancer in northern Iran. Cell Mol Biol (Noisy-le-grand) 2015;61:21–4. 26255264

[17] Jing B Wang J Chang WL. Association of the polymorphism of APE1gene with the risk of prostate cancer in Chinese Han population. Clinical Lab 2013;59:163–8. 10.7754/clin.lab.2012.120206 23505922

[18] Kuasne H Rodrigues IS Losi-Guembarovski R. Base excision repair genes XRCC1 and APEX1 and the risk for prostate cancer. Mol Biol Rep 2011;38:1585–91. 20852942 10.1007/s11033-010-0267-z

[19] Lan C Ambrosone CB Lee J. Association between polymorphisms in the DNA repair genes XRCC1 and APE1, and the risk of prostate cancer in white and black Americans. J Urology 2006;175:108–12. 10.1016/S0022-5347(05)00042-X 16406883

[20] Lavender NA Komolafe OO Benford M. No association between variant DNA repair genes and prostate cancer risk among men of African descent. Prostate 2010;70:113–9. 19760636 10.1002/pros.21048 PMC2798907

[21] Mandal RK Gangwar R Kapoor R. Polymorphisms in base-excision & nucleotide-excision repair genes & prostate cancer risk in north Indian population. Indian J Med Res 2012;135:64–71. 22382185 10.4103/0971-5916.93426 PMC3307187

[22] Mittal RD Mandal RK Gangwar R. Base excision repair pathway genes polymorphism in prostate and bladder cancer risk in North Indian population. Mech Ageing Dev 2012;133:127–32. 22019847 10.1016/j.mad.2011.10.002

[23] Poirier MC Weston A. Bertino JR. DNA damage, DNA repair, and mutagenesis. Encyclopedia of cancer. Boston, MA: Academic Press; 2002. 79–82.

[24] Martin FL Cole KJ Muir GH. Primary cultures of prostate cells and their ability to activate carcinogens. Prostate Cancer Prostatic Dis 2002;5:96–104. 12496996 10.1038/sj.pcan.4500579

[25] Osipov M Sokolnikov M. Previous malignancy as a risk factor for the second solid cancer in a cohort of nuclear workers. PLOS One 2021;3:08–15.

[26] Korançe F. The growing relation between environment and public health. SciMed J 2021;3:100–15.

[27] Mohrenweiser HW Wilson DM 3rd Jones IM. Challenges and complexities in estimating both the functional impact and the disease risk associated with the extensive genetic variation in human DNA repair genes. Mutat Res 2003;526:93–125. 12714187 10.1016/s0027-5107(03)00049-6

[28] Chen DS Herman T Demple B. Two distinct human DNA diesterases that hydrolyze 3V-blocking deoxyribose fragments from oxidized DNA. Nucleic Acids Res 1991;9:5907–14. 10.1093/nar/19.21.5907 PMC329046 1719484

[29] Hu JJ Smith TR Miller MS. Genetic regulation of ionizing radiation sensitivity and breast cancer risk. Environ Mol Mutagen 2002;39:208–15. 11921191 10.1002/em.10058

[30] Lo YL Jou YS Hsiao CF. A polymorphism in the APE1 gene promoter is associated with lung cancer risk. Cancer Epidemiol Biomarkers Prev 2009;18:223–9. 19124501 10.1158/1055-9965.EPI-08-0749

[31] Wang M Chu H Wang S. Genetic variant in APE1 gene promoter contributes to cervical cancer risk. Am J Obstet Gynecol 2013;209:360.e1–7. 23871947 10.1016/j.ajog.2013.07.010

[32] Lu J Zhang S Chen D. Functional characterization of a promoter polymorphism in APE1/Ref-1 that contributes to reduced lung cancer susceptibility. FASEB J 2009;23:3459–69. 19541747 10.1096/fj.09-136549

[33] Kang H Dai Z Ma X. A genetic variant in the promoter of APE1 gene (-656 T>G) is associated with breast cancer risk and progression in a Chinese population. Gene 2013;531:97–100. 23994194 10.1016/j.gene.2013.08.052

[34] Jing B Wang J Chang WL. Association of the polymorphism of APE1 gene with the risk of prostate cancer in Chinese Han population. Clin Lab 2013;59:163–8. 23505922 10.7754/clin.lab.2012.120206

[35] Zhao Q Wang W Zhang Z. A genetic variation in APE1 is associated with gastric cancer survival in a Chinese population. Cancer Sci 2011;102:1293–7. 21615620 10.1111/j.1349-7006.2011.01959.x

[36] Demple B Harrison L. Repair of oxidative damage to DNA: enzymology and biology. Ann Rev Biochem 1994;63:915–48. 7979257 10.1146/annurev.bi.63.070194.004411

[37] Evans AR Limp-Foster M Kelley MR. Going APE over ref-1. Mutat Res 2000;461:83–108. 11018583 10.1016/s0921-8777(00)00046-x

[38] Gu D Wang M Zhang Z. The DNA repair gene APE1 T1349G polymorphism and cancer risk: a meta-analysis of 27 case-control studies. Mutagenesis 2009;24:507–12. 19762350 10.1093/mutage/gep036

[39] Hu JJ Smith TR Miller MS. Amino acid substitution variants of APE1 and XRCC1 genes associated with ionizing radiation sensitivity. Carcinogenesis 2001;22:917–22. 11375899 10.1093/carcin/22.6.917

[40] Kelley MR Cheng L Foster R. Elevated and altered expression of the multifunctional DNA base excision repair and redox enzyme Ape1/ref-1 in prostate cancer. Clin Cancer Res 2001;7:824–30. 11309329

[41] Gu D Wang M Wang M. The DNA repair gene APE1 T1349G polymorphism and cancer risk: a meta-analysis of 27 case-control studies. Mutagenesis 2009;24:507–12. 19762350 10.1093/mutage/gep036

